# EQ-5D-5 L utilities per health states in Spanish population with knee or hip osteoarthritis

**DOI:** 10.1186/s12955-019-1230-x

**Published:** 2019-10-30

**Authors:** Lidia García-Pérez, Vanesa Ramos-García, Pedro Serrano-Aguilar, José Luis Pais-Brito, María Aciego de Mendoza, Jesús Martín-Fernández, Roberto García-Maroto, Juan Carlos Arenaza, Amaia Bilbao

**Affiliations:** 1 Fundación Canaria de Investigación Sanitaria (FUNCANIS), Camino Candelaria No 44, 1a planta. 38109 El Rosario, Santa Cruz de Tenerife, Spain; 2 Servicio de Evaluación del Servicio Canario de la Salud (SESCS), Camino Candelaria No 44, 1a planta. 38109 El Rosario, Santa Cruz de Tenerife, Spain; 3 Health Services Research on Chronic Patients Network (REDISSEC), Bilbao, Spain; 4 Instituto Universitario de Desarrollo Regional (IUDR), University of La Laguna, San Cristóbal de La Laguna, Spain; 5 Centro de Investigaciones Biomédicas de Canarias (CIBICAN), San Cristóbal de La Laguna, Spain; 6 Department of Orthopaedics and Traumatology, University Hospital of Canary Islands, Ctra. Ofra S/N La Cuesta, 38320 La Laguna, Tenerife, Spain; 7 Department of Orthopaedics and Traumatology, University of La Laguna, San Cristóbal de La Laguna, Spain; 8 Multiprofessional Teaching Unit of Primary and Community Care “Oeste”, Health Service, c/ Alonso Cano 8, Móstoles, 28933 Madrid, Madrid Spain; 9 Health Sciences Faculty, Rey Juan Carlos University, Madrid, Spain; 10 Traumatology and Orthopedic Surgery Service, Hospital Universitario Clínico San Carlos, C/ Profesor Martín Lagos S/N, 28040 Madrid, Spain; 11 Traumatology and Orthopedic Surgery Service, Basurto University Hospital (Osakidetza), Avda. Montevideo, 18, 48013 Bilbao, Bizkaia Spain; 12 Research Unit, Basurto University Hospital (Osakidetza), Avda. Montevideo, 18, 48013 Bilbao, Bizkaia Spain

**Keywords:** EQ-5D-5L, Health states, Hip osteoarthritis, Knee osteoarthritis, Utilities

## Abstract

**Background:**

The objective of this study was to obtain utilities by means of EQ-5D-5L for different health states in patients with knee osteoarthritis (KOA) or hip osteoarthritis (HOA) in Spain, and to compare these values with those used in foreign studies with the aim of discussing their transferability for their use in economic evaluations conducted in Spain.

**Methods:**

Primary study: Observational prospective study of KOA or HOA patients in Spain. Sociodemographic and clinical characteristics were collected to characterize the sample. Utilities were elicited using the EQ-5D-5L questionnaire. ANOVA and bivariable analyses were conducted to identify differences between health states. Literature review: Using the bibliographic databases NSH EED and CEA Registry, we conducted searches of model-based cost utilities analyses of technologies in KOA or HOA patients. Health states and utilities were extracted and compared with values obtained from the Spanish sample.

**Results:**

Three hundred ninety-seven subjects with KOA and 361 subjects with HOA were included, with average utilities of 0.544 and 0.520, respectively. In both samples, differences were found in utilities according to level of pain, stiffness and physical function (WOMAC) and severity of symptoms (Oxford scales), so that the worst the symptoms, the lower the utilities. The utilities after surgery were higher than before surgery. Due to limitations from our study related to sample size and observational design, it was not possible to estimate utilities for approximately half the health states included in the published models because they were directly related to specific technologies. For almost 100% of health states of the selected studies we obtained very different utilities from those reported in the literature.

**Conclusions:**

To our knowledge this is the first article with detailed utilities estimated using the EQ-5D-5L in Spain for KOA and HOA patients. In both populations, utilities are lower for worse health states in terms of level of pain, stiffness and physical function according to WOMAC, and according to the Oxford scales. Most utilities obtained from the Spanish sample are lower than those reported in the international literature. Further studies estimating utilities from local populations are required to avoid the use of foreign sources in economic evaluations.

## Background

Osteoarthritis causes pain and functional incapacity. In developed societies with a high life expectancy, the prevalence of knee osteoarthritis (KOA) and hip osteoarthritis (HOA) is high, 23.9 and 10.9%, respectively [1]. Osteoarthritis entails a social and economic burden in terms of health-related quality of life (HRQoL) [2, 3] and cost of the disease [2, 4]. Estimated expenses for KOA or HOA are equal to 0.5% of Spain’s GDP [5].

Health problems with such an impact on society and the existent technologies for those health problems deserve to be the focus of health technology assessment and economic evaluation specifically to inform the evidence-based decision making by health authorities. During an economic evaluation it is common to conduct cost-utility analysis (CUA), that is, a comparison of at least two alternatives in terms of costs and outcomes where the outcome measure is expressed as quality-adjusted life years (QALY). The adjustment of quantity of life for quality of life is attained by means of application of weightings that try to reflect the desirability of different states of health by individuals or society and that are denominated utilities or health state utilities [6, 7]. For example, 1 year lived in perfect life (utility = 1) implies 1 QALY, but 1 year lived in a less perfect situation (utility = 0.5, for example) implies less than one QALY (0.5 QALYs in this case). The main advantage of using QALYs as a measurement is due to its potentiality to be used to compare different technologies and even different diseases [6]. Moreover, cost-effectiveness thresholds have been estimated and/or set up in different countries to be used as a limit to decide those technologies to be reimbursed or included in the health care systems based on their cost-effectiveness [8]. For example, in Spain the most recent estimated threshold is 25,000 €/QALY, so any new technology with an incremental cost-effectiveness ratio over this threshold should not be adopted according to this study [9].

The EQ-5D [10], a generic HRQoL questionnaire, is the tool most commonly used in Spain to measure utilities [11]. In its most current version, [12] the EQ-5D-5 L questionnaire consists of two sections: a visual analogue scale to evaluate HRQoL from 0 to 100 and a questionnaire comprised of 5 questions or dimensions (mobility, self-care, performing of usual activities, pain/discomfort and anxiety/depression) with 5 response levels (from no problems to extreme problems). Using combinations of these five questions it can be obtained 3125 (5^5^) health states and a weighted health score denominated utility index associated with each health state. This index varies from 1 (perfect health) to negative values (0 being the value equivalent to death) because valuation studies have found that there are less preferred states than death by the general population. The social value set obtained from the general population in Spain for the EQ-5D-5L has recently been published [13]. A previous article has analyzed the psychometric properties of the EQ-5D-5 L in osteoarthritis patients in Spain [14]: reliability (Cronbach’s alpha = 0.86), validity (Spearman’s correlation coefficient of EQ-5D-5 L index and WOMAC pain and function subscales: − 0.688 and − 0.782, respectively) and responsiveness (floor and ceiling effects < 3%).

In a systematic review of utilities obtained from the Spanish population [11] it was found that 94% of articles used the EQ-5D questionnaire and that health state utilities for a significant number of diseases are unknown. The highest number of utilities was collated in the hospital and specialized care settings whilst only 18% of utilities were collected in primary care [11]. This same review identified three primary papers reporting utility values obtained from the Spanish population with KOA or HOA [15–17]. The values varied from 0.2 in patients with total knee replacement (TKR) before surgery to 0.64 at 6 months from surgery [15].

The economic evaluation of technologies applied to osteoarthritis, be they surgical [18, 19], pharmacologic [20] or other interventions [21], is of interest for researchers, professionals and health policy decision makers [22]. In a systematic review of CUA performed in Spain [23] three studies that evaluated technologies in KOA or HOA patients were identified [24–26]. None of these studies used health state utilities obtained from the Spanish population, but from studies conducted in other countries.

The lack of transferability of economic evaluations between countries makes necessary to perform studies in the local population [27]. Moreover, utilities used in economic evaluations ideally would proceed from studies performed in the local population or countries with similar context although very often values from foreign populations found in a review of literature is the only available source [7, 28]. The ultimate purpose of this study is determining the values of utilities for a broad variety of health states obtained from Spanish population with KOA or HOA with different level of severity and being treated in different health care settings so that they are usable for future economic evaluations in Spain. Consequently two aims were pursued: 1) obtaining health state utilities in patients with knee or hip osteoarthritis using an observational study performed in Spain, and 2) comparing these values with others obtained in international studies with the purpose of discussing the transferability of utilities in KOA and HOA subjects.

## Methods

To achieve these two aims, first, data collected in a primary study with observational and prospective design were analyzed. Second, a review of health state utilities included in published economic evaluations was performed.

### Primary study

#### Participants

An opportunistic and consecutive sample was recruited between January and December 2015 by doctors in traumatology, rheumatology and primary care consultations from three different areas of Spain (Vizcaya, Madrid and Tenerife). To be included patients had to be adults (> 18 years), diagnosed of KOA or HOA according to criteria of the American College of Rheumatology [29] independently of the severity of the disease, and agreed to participate after being informed. Excluded were all those patients who did not understand Spanish, did not know how to read or who suffered from diagnosed cognitive impairment.

Sample size was estimated to achieve other objectives pursued in the project that required more power (mapping between EQ-5D-5 L and clinical questionnaires) than the descriptive analysis presented here; more information elsewhere [14, 30, 31]. It was estimated that 360 KOA or HOA patients were necessary. Assuming a loss rate of 10% for incomplete data based on prior experiences and a 75% response rate, recruitment of 712 subjects was set as an aim. In the end we were able to recruit more than needed, 758 patients with complete baseline data. Subjects were included in the study after giving their informed consent. The study received approval by the Ethics Committees for Clinical Research from the three geographical areas.

#### Variables

Data were collected at the time of recruitment and at 6 months both by the clinician and the patient. Sociodemographic variables (age, sex, region of residency, education, marital status and work situation) and clinical variables: comorbidity measured using the Charlson Index [32], weight, height, body mass index (BMI), joints affected by arthrosis, time since diagnosis, treatments received (pharmacologic, surgery, rehabilitation and physiotherapy) and healthcare setting where the patient was recruited, were included. Self-perceived measures were: Oxford Knee Score (OKS) [33] or Oxford Hip Score (OHS) [34]; Western Ontario and McMaster Universities Osteoarthritis Index (WOMAC) [35]; and the EQ-5D-5 L questionnaire [12] applying the value set published in Spain [13].

The OKS and OHS instruments measure the severity of symptoms in patients with KOA and HOA, respectively [33, 34]. They are comprised of 12 questions and the score varies from 0 to 48 where 0 is the worst and 48 the best such that patients can be classified into 4 groups: satisfactory joint function (40–48), mild to moderate arthritis (30–39), moderate to severe arthritis (20–29), severe arthritis (0–19) [36]. These questionnaires were recently validated in the Spanish population [30, 31].

The WOMAC is a multidimensional scale comprised of 24 items that measures dimensions: pain (5 items), stiffness (2 items) and physical function (17 items) in osteoarthritis patients [35]. This study used the Likert version with 5 answer levels for each item, which represent different degrees of intensity (none, mild, moderate, severe or extreme) graded from 0 to 4. This score is added and standardized from 0 to 100 such that the higher the score the worse the patient’s condition. This questionnaire was validated in Spain for KOA and HOA patients [37].

#### Statistical analysis

Sample characteristics are reported as mean and standard deviation (SD) and frequencies and percentages for continuous and categorical variables, respectively. The proportion of patients by dimension and level of response in the EQ-5D-5 L questionnaire are presented. Subgroups were created using clinical baseline and self-reported information for the variables mentioned above (WOMAC, OKS, OHS, number of comorbidities, BMI, etc.). Although some of the subgroups are not necessarily associated to a health condition, such as age group, these subgroups are used for the estimation of the corresponding utilities and we call them indistinctly health states or subgroups. For those patients who underwent a surgical procedure during the 6 months follow-up health states are defined and utilities estimated using a follow-up questionnaire that included the same questions used for the baseline. In both cases the mean and SD for health state utilities are reported. Results were compared with normative values obtained from general population interviewed in the Spanish National Health Survey (2011–2012) [38]. We hypothesized that different (worse) health states mean different (lower) utilities. To identify statistically significant differences between subgroups, ANOVA and student *t* analyses with multiple testing correction were performed (Bonferroni test when we assume population variances are equal after Levene’s test or Tamhane’s T2 in other cases). *P* < 0.05 was considered statistically significant; *P* < 0.01 for multiple comparisons. The program IBM SPSS Statistics 24.0.0.1 (Armonk, NY; IBM Corp) was used to perform the statistical analyses.

### Literature review

A systematic search was performed in July 2017 in the NSH EED database (Center for Reviews and Dissemination, University of York). The search strategy is included as Additional file 1: Table S1. Reviewing the title and abstract, those economic evaluations based on models that evaluated technologies in KOA or HOA patients and that included QALYs among the outcome measures were selected. The CEA Registry (Cost-Effectiveness Analysis Registry) database was used [39] to extract health states and health state utilities included in these economic evaluations. The CEA Registry is a comprehensive database of > 7000 cost-utility analyses from literature published in English around the world where utility values, health states and costs per QALY are collected [39]. In addition, papers selected were reviewed and information on the origin and kind of instrument used to obtain utilities was extracted.

Health states equivalent to those health states identified in the literature review were defined using our data, and utilities from our sample of Spanish patients were obtained according to the methodology reported above. An analysis of the utilities obtained with our sample was performed in comparison to utilities reported in the international literature. According to McClure et al., the minimally relevant difference in utilities for EQ-5D-5 L for Spain was estimated at 0.045 [40], whereby we assessed differences identified with this value.

## Results

Statistical differences were found between the utility index for the sample with a diagnosis of KOA or HOA (mean = 0.533; *N* = 750) and the normative value obtained for the Spanish general population (mean = 0.896; *N* = 20,560) (*P* < 0.0001). Differences were also found for men, women, and each age group except for the population older than 85 years (*P* = 0.630) (Additional file 1: Table S2).

### Results of the primary study

Knee osteoarthritis: A total of 397 subjects were recruited with KOA (Table 1). Average age was 71.42 years (SD: 9.06) (range: 35–94) and 70% were women. A total of 58% of the sample was recruited in primary care health centers, the remainder were recruited in hospital; 42% have arthrosis in both knees. Average scores of self-administered questionnaires, WOMAC, OKS and EQ-5D-5 L index were respectively, 49.63 (SD: 20.32), 21.96 (SD: 9.96), 0.544 (SD: 0.271).

**Table 1:** Characteristics of the samples with knee and hip osteoarthritis

Characteristic		Knee osteoarthritis				Hip osteoarthritis			
		N	%	Mean	SD	N	%	Mean	SD
Age (years)		397		71.42	9.06	361		67.88	11.67
Sex	Women	277	70			192	53		
	Men	120	30			169	47		
Region	Basque Country	158	40			157	44		
	Canary Islands	81	20			80	22		
	Madrid	158	40			124	34		
Type of health center	Primary healthcare center	230	58			204	57		
	Hospital	167	42			157	44		
Joint with osteoarthritis	Right	110	28			138	38		
	Left	119	30			121	34		
	Both joints	168	42			102	28		
BMI		389		29.67	4.87	355		28.16	4.57
Weight (kg)		395		77.67	14.00	357		76.47	15.04
Height (cm)		389		161.93	8.57	356		164.57	8.84
Charlson Index (No of comorbidities)		395		0.78	1.14	361		0.84	1.31
WOMAC – Pain score (0–100)		396		47.00	20.49	360		45.81	22.77
WOMAC – Stiffness score (0–100)		396		46.81	25.42	360		48.26	26.05
WOMAC – Physical function score (0–100)		397		51.08	20.95	360		52.43	23.05
WOMAC Score (0–100)		397		49.63	20.32	359		50.69	22.25
Oxford Scale Score (0–48)		395		21.96	9.96	359		22.84	10.62
EQ-5D-5 L Index		393		0.544	0.271	357		0.520	0.304
EQ-5D VAS (0–100)		390		57.36	21.55	359		54.47	22.27

*BMI* Body mass index, *SD* Standard deviation, *WOMAC* Western Ontario and McMaster Universities Osteoarthritis Index

WOMAC: 0, worst; 100, best

Oxford Scale Score: 0, worst; 48, best (severity of arthritis)

EQ-5D-5 L Index: 0, death; 1, perfect health

The most common state of health (32331) corresponds to a patient with moderate problems in the dimensions mobility, daily activities and pain/discomfort, slight problems in self-care and not anxious or depressed. The second most common state of health (11121) corresponds to a patient with slight pain or discomfort and no problems in the other dimensions (Additional file 1: Table S3). By dimensions the moderate level was the most common in mobility (45.8%), usual activities (36.3%) and pain/discomfort (38.5%). Self-care level 1 (no problems) and level 3 (moderate) were reported equally (32%). For anxiety/depression 43.3% of the sample reported not having any problems. Levels 4 and 5 (severe and extreme) were reported less commonly although 30.2% of patients reported having severe pain or discomfort and 24.4% reported severe problems in mobility (Table 2). Among the 18 patients that reported negative utility values, the most common health state was 44444 (4 patients), that is, with severe problems in the five dimensions.

**Table 2:** Percentage of patients with knee osteoarthritis reporting levels within EQ-5D-5 L dimensions

Group	Dimension	Level 1	Level 2	Level 3	Level 4	Level 5
The whole sample (*N* = 397)	Mobility	11.8	16.6	**45.8**	24.4	1.3
	Self-care	**32.0**	27.0	**32.0**	8.8	0
	Usual activities	16.6	26.7	**36.3**	15.9	4.3
	Pain/Discomfort	4.0	23.2	**38.5**	30.2	4.0
	Anxiety/Depression	**43.3**	24.2	18.1	10.8	2.5
OKS – severe (*N* = 168)	Mobility	2.4	7.1	36.9	**50.6**	3.0
	Self-care	10.1	18.5	**51.8**	19.6	0
	Usual activities	2.4	10.1	**44.6**	33.9	8.9
	Pain/Discomfort	1.2	3.0	33.9	**52.4**	9.5
	Anxiety/Depression	**28.0**	23.2	25.6	17.9	5.4
OKS - moderate to severe (*N* = 128)	Mobility	5.5	17.2	**69.5**	7.8	0
	Self-care	28.1	**43.0**	27.3	1.6	0
	Usual activities	10.9	36.7	**46.1**	4.7	1.6
	Pain/Discomfort	0.8	23.4	**51.6**	24.2	0
	Anxiety/Depression	**44.5**	25.8	20.3	9.4	0
OKS - mild to moderate (*N* = 74)	Mobility	29.7	**35.1**	**35.1**	0	0
	Self-care	**68.9**	28.4	2.7	0	0
	Usual activities	39.2	**51.4**	9.5	0	0
	Pain/Discomfort	10.8	**55.4**	32.4	1.4	0
	Anxiety/Depression	**67.6**	27.0	2.7	1.4	1.4
OKS - satisfactory joint function (*N* = 21)	Mobility	**66.7**	19.0	14.3	0	0
	Self-care	**100**	0	0	0	0
	Usual activities	**85.7**	4.8	9.5	0	0
	Pain/Discomfort	19.0	**66.7**	14.3	0	0
	Anxiety/Depression	**81.0**	14.3	4.8	0	0

Level 1: indicating no problem; Level 2: indicating slight problems; Level 3: indicating moderate problems; Level 4: indicating severe problems; Level 5: indicating extreme problems

Highest frequencies are highlighted in bold letters

Table 3 shows the health state utilities for different population subgroups. No statistically significant differences were detected between groups according to age, sex, region of Spain, joint with osteoarthritis (right or left), time since diagnosis (years), with or without prosthesis in the other joint and number of comorbidities. Differences identified between BMI categories (*P* = 0.029; ANOVA) disappeared when multiple comparison tests were conducted. Regarding clinical questionnaires (WOMAC, OKS), differences were found between all subgroups (the less clinical problems, the higher utilities) except for those two with less joint stiffness according to WOMAC (*P* = 0.389; Tamhane’s T2). There was no difference between receiving and not receiving any pharmacologic treatment (*P* = 0.321), although patients taking opioid pain medication had significantly lower utilities than the whole sample (*P* = 0.005). During the 6-month follow up, 65 people out of 92 waiting for a prosthesis had had a TKR. There was an increase in the utility index after the TKR (*P* < 0.0001).

**Table 3:** Utility weights (EQ-5D-5 L Index) per subgroup in patients with knee osteoarthritis

Subgroup		N	Mean	SD	*P*-value
Age groups (years)	< 45	1			0.746
	45–54.99	17	0.516	0.302	
	55–64.99	71	0.540	0.269	
	65–74.99	143	0.564	0.279	
	75–84.99	143	0.523	0.264	
	≥85	18	0.589	0.258	
Gender	Women	275	0.528	0.269	0.079
	Men	118	0.581	0.274	
Region	Basque Country	154	0.503	0.570	0.053
	Madrid	158	0.572	0.252	
	Canary Islands	81	0.568	0.270	
BMI categories	Underweight^a^	0	–	–	0.029
	Normal^b^	61	0.590	0.214	
	Overweight^c^	154	0.577	0.261	
	Obesity^d^	161	0.500	0.299	
	Morbid obesity^e^	10	0.465	0.215	
Joint with osteoarthritis	Right knee	109	0.559	0.261	0.752
	Left knee	117	0.546	0.259	
	Both knees	167	0.533	0.287	
Time since osteoarthritis diagnosis (years)	<1	62	0.602	0.245	0.188
	1 to < 5	181	0.547	0.269	
	5 to < 10	93	0.536	0.270	
	10 to < 15	35	0.466	0.307	
	≥15	21	0.513	0.297	
Contralateral knee	Without prosthesis in the other knee	314	0.546	0.275	0.986
	With prosthesis in the other knee	78	0.545	0.250	
Other joints	Hip osteoarthritis	53	0.519	0.312	0.101
	No hip osteoarthritis	339	0.549	0.265	
	Osteoarthritis in other joints	228	0.535	0.273	0.813
	No osteoarthritis in other joints	164	0.558	0.269	
Number of comorbidities (Charlson Index)	No comorbidity	215	0.572	0.257	0.149
	1 comorbidity	104	0.523	0.282	
	2 comorbidities	40	0.504	0.301	
	3 comorbidities	18	0.435	0.279	
	≥4 comorbidities	14	0.522	0.282	
WOMAC	Pain score 0 to <25^a^	42	0.783^bcd^	0.187	< 0.0001
	Pain score 25 to < 50^b^	163	0.648^acd^	0.186	
	Pain score 50 to <75^c^	139	0.450^abd^	0.248	
	Pain score 75 to 100^d^	48	0.252^abc^	0.283	
	Stiffness score 0 to <25^a^	44	0.716^cd^	0.207	< 0.0001
	Stiffness score 25 to < 50^b^	134	0.652^cd^	0.215	
	Stiffness score 50 to <75^c^	127	0.506^abd^	0.250	
	Stiffness score 75 to 100^d^	87	0.347^abc^	0.277	
	Physical function score 0 to <25^a^	41	0.845^bcd^	0.113	< 0.0001
	Physical function score 25 to <50^b^	146	0.664^acd^	0.173	
	Physical function score 50 to <75^c^	143	0.475^abd^	0.205	
	Physical function score 75 to 100^d^	63	0.230^abc^	0.293	
	WOMAC Score 0 to <25^a^	44	0.829^bcd^	0.117	< 0.0001
	WOMAC Score 25 to <50^b^	145	0.665^acd^	0.172	
	WOMAC Score 50 to <75^c^	143	0.464^abd^	0.220	
	WOMAC Score 75 to 100^d^	50	0.189^abc^	0.277	
Oxford Knee Score (OKS)	Severe (0–19)^a^	168	0.346^bcd^	0.255	< 0.0001
	Moderate to severe (20–29)^b^	128	0.609^acd^	0.157	
	Mild to moderate (30–39)^c^	74	0.780^abd^	0.109	
	Satisfactory joint function (40–48)^d^	21	0.879^abc^	0.097	
Non-surgery treatments	No pharmacologic treatment	53	0.579	0.299	0.321
	Any pharmacologic treatment	340	0.539	0.267	
	Symptomatic slow acting drugs for osteoarthritis (SYSADOA)	50	0.564	0.247	0.567*
	Non-opioid pain medication	242	0.546	0.263	0.919*
	Nonsteroidal anti-inflammatory drugs (NSAID)	152	0.529	0.283	0.529*
	Opiate derivatives	96	0.538	0.274	0.836*
	Opioid pain medication	21	0.333	0.309	0.005*
	Rehabilitation / Physiotherapy	14	0.559	0.295	0.854*
Knee replacement	Waiting for prosthesis	92	0.400	0.266	< 0.0001**
	Prosthesis in the last 6 months	65	0.683	0.243	
	Complication after prosthesis	2	0.424	0.007	–

*BMI* Body mass index, *SD* Standard deviation, *WOMAC* Western Ontario and McMaster Universities Osteoarthritis Index

*p*-values represents the significance of differences between groups by means of ANOVA except for (*) *P*-value comparing with the whole sample’s utility value (0.544) (one sample *t*-test), and (**) *P*-value comparing sample before and after (6 months) the prosthesis (paired sample *t*-test)

Superscript letters indicate differences among the subgroups according to Tamhane’s T2 post hoc test for multiple comparisons at *P*-value< 0.01

Hip osteoarthritis: A total of 361 subjects were recruited with HOA (Table 1). Average age was 67.88 years (SD: 11.67) (range: 36–89) and 53% were women. A total of 57% of the sample was recruited in primary care health centers, the remainder were recruited in hospital. A total of 28% have osteoarthritis in both hips. Average scores of self-administered questionnaires, WOMAC, OHS and EQ-5D-5 L Index, were respectively, 50.69 (SD: 22.25), 22.84 (SD: 22.25), and 0.520 (SD: 0.304).

The most common health state (11111) corresponds to a patient with perfect health. The second most common health state (33331) corresponds to a patient with moderate problems in all dimensions except anxiety/depression where there are no problems (Additional file 1: Table S3). By dimensions, the most common moderate level was for mobility (39.3%), self-care (31.3%), usual activities (37.1%) and pain/discomfort (34.9%). For anxiety/depression 43.2% of the sample reported not having any problems. Levels 4 and 5 (severe and extreme) were reported less commonly although 30.2% of patients reported having severe pain or discomfort and 23.3% reported severe problems in mobility (Table 4). Among the 26 patients that reported negative utility values, the most common health state was 44444 (6 patients).

**Table 4:** Percentage of patients with hip osteoarthritis reporting levels within EQ-5D-5 L dimensions

Group	Dimension	Level 1	Level 2	Level 3	Level 4	Level 5
The whole sample (*N* = 361)	Mobility	10.5	23.8	**39.3**	23.3	2.8
	Self-care	25.2	27.7	**31.3**	13.6	1.9
	Usual activities	16.6	23.0	**37.1**	17.2	5.8
	Pain/Discomfort	8.3	20.2	**34.9**	30.2	6.1
	Anxiety/Depression	**43.2**	23.2	18.0	10.8	3.9
OHS – severe (*N* = 149)	Mobility	0.7	3.4	38.9	**50.3**	6.7
	Self-care	4.0	12.8	**49.0**	30.2	4.0
	Usual activities	2.0	5.4	**43.6**	36.9	12.1
	Pain/Discomfort	0	4.0	24.2	**58.4**	13.4
	Anxiety/Depression	21.5	19.5	**31.5**	18.8	8.7
OHS - moderate to severe (*N* = 116)	Mobility	3.4	32.8	**56.9**	6.9	0
	Self-care	20.7	**44.0**	32.8	2.6	0
	Usual activities	8.6	36.2	**47.4**	6.0	1.7
	Pain/Discomfort	1.7	21.6	**57.8**	18.1	0.9
	Anxiety/Depression	**43.1**	35.3	11.2	9.5	0.9
OHS - mild to moderate (*N* = 61)	Mobility	26.2	**50.8**	23.0	0	0
	Self-care	**55.7**	39.3	3.3	1.6	0
	Usual activities	39.3	**42.6**	18.0	0	0
	Pain/Discomfort	16.4	**52.5**	29.5	1.6	0
	Anxiety/Depression	**73.8**	18.0	8.2	0	0
OHS - satisfactory joint function (*N* = 29)	Mobility	**58.6**	27.6	13.8	0	0
	Self-care	**86.2**	13.8	0	0	0
	Usual activities	**72.4**	20.7	6.9	0	0
	Pain/Discomfort	**58.6**	31.0	10.3	0	0
	Anxiety/Depression	**93.1**	6.9	0	0	0

Level 1: indicating no problem; Level 2: indicating slight problems; Level 3: indicating moderate problems; Level 4: indicating severe problems; Level 5: indicating extreme problems

Highest frequencies are highlighted in bold letters

Table 5 shows the health state utilities for different population subgroups. No statistically significant differences were detected between groups according to age, sex, BMI, joint with osteoarthritis (right or left), time since diagnosis (years), with or without prosthesis in the other joint. Differences identified depending on region of residence and number of comorbidities (*P* < 0.0001; ANOVA) disappeared when multiple comparison tests were conducted. Regarding clinical questionnaires (level of pain, stiffness and physical function according to WOMAC, and OHS), differences were found between all subgroups (*P* < 0.0001), that is, the less joint problems, the higher utilities. For example, utilities varied according to the degree of severity using the OHS from of 0.269 (severe) to 0.903 (satisfactory function). There was no difference between receiving and not receiving any pharmacologic treatment (*P* = 0.321). During the 6-month follow up, 65 out of 97 people waiting for a prosthesis had the THR. There was an increase in the utility index after the THR (*P* < 0.0001).

**Table 5:** Utility weights (EQ-5D-5 L Index) per subgroup in patients with hip osteoarthritis

Subgroup		N	Mean	SD	*P*-value
Age groups (years)	< 45	13	0.591	0.357	0.259
	45–54.99	37	0.506	0.291	
	55–64.99	83	0.465	0.291	
	65–74.99	101	0.507	0.298	
	75–84.99	103	0.562	0.299	
	≥85	20	0.584	0.380	
Gender	Women	188	0.512	0.305	0.606
	Men	169	0.529	0.304	
Region	Basque Country^a^	154	0.473	0.313	0.036
	Madrid^b^	124	0.553	0.288	
	Canary Islands^c^	79	0.562	0.301	
BMI categories	Underweight	2	0.339	0.572	0.372
	Normal	87	0.552	0.305	
	Overweight	154	0.530	0.288	
	Obesity	102	0.473	0.324	
	Morbid obesity	5	0.541	0.241	
Joint with osteoarthritis	Right hip	135	0.481	0.320	0.059
	Left hip	121	0.571	0.285	
	Both hip	101	0.511	0.297	
Time since osteoarthritis diagnosis (years)	<1	118	0.541	0.279	0.240
	1 to <5	152	0.492	0.309	
	5 to < 10	63	0.520	0.347	
	10 to < 15	14	0.675	0.204	
	≥15	8	0.523	0.288	
Contralateral hip	Without prosthesis in the other hip	293	0.510	0.303	0.182
	With prosthesis in the other hip	62	0.567	0.302	
Other joints	Knee osteoarthritis	115	0.522	0.322	0.303
	No knee osteoarthritis	241	0.521	0.295	
	Osteoarthritis in other joints	214	0.537	0.301	0.801
	No osteoarthritis in other joints	142	0.499	0.306	
Number of comorbidities (Charlson Index)	No comorbidity^a^	206	0.561	0.284	0.033
	1 comorbidity^b^	71	0.465	0.323	
	2 comorbidities^c^	46	0.495	0.334	
	3 comorbidities^d^	18	0.386	0.370	
	≥4 comorbidities^e^	16	0.460	0.210	
WOMAC	Pain score 0 to <25^a^	54	0.818^bcd^	0.146	< 0.0001
	Pain score 25 to <50^b^	143	0.643^acd^	0.175	
	Pain score 50 to <75^c^	110	0.414^abd^	0.243	
	Pain score 75 to 100^d^	49	0.074^abc^	0.250	
	Stiffness score 0 to <25^a^	42	0.795^bcd^	0.181	< 0.0001
	Stiffness score 25 to <50^b^	107	0.656^acd^	0.220	
	Stiffness score 50 to <75^c^	118	0.509^abd^	0.241	
	Stiffness score 75 to 100^d^	89	0.243^abc^	0.294	
	Physical function score 0 to <25^a^	48	0.878^bcd^	0.098	< 0.0001
	Physical function score 25 to <50^b^	115	0.672^acd^	0.144	
	Physical function score 50 to <75^c^	125	0.464^abd^	0.225	
	Physical function score 75 to 100^d^	69	0.120^abc^	0.229	
	WOMAC Score 0 to <25^a^	47	0.864^bcd^	0.122	< 0.0001
	WOMAC Score 25 to <50^b^	124	0.673^acd^	0.145	
	WOMAC Score 50 to <75^c^	120	0.429^abd^	0.232	
	WOMAC Score 75 to 100^d^	56	0.081^abc^	0.222	
Oxford Hip Score (OHS)	Severe (0–19)^a^	149	0.269^bcd^	0.264	< 0.0001
	Moderate to severe (20–29)^b^	116	0.611^acd^	0.146	
	Mild to moderate (30–39)^c^	61	0.783^abd^	0.101	
	Satisfactory joint function (40–48)^d^	29	0.903^abc^	0.102	
Non-surgery treatments	No pharmacologic treatment	69	0.544	0.312	0.491
	Any pharmacologic treatment	287	0.516	0.301	
	Symptomatic slow acting drugs for osteoarthritis (SYSADOA)	34	0.574	0.320	0.331*
	Non-opioid pain medication	197	0.503	0.309	0.435*
	Nonsteroidal anti-inflammatory drugs (NSAID)	128	0.533	0.304	0.613*
	Opiate derivatives	97	0.498	0.304	0.474*
	Opioid pain medication	13	0.435	0.378	0.434*
	Rehabilitation / Physiotherapy	10	0.598	0.227	0.305*
Hip replacement	Waiting for prosthesis	97	0.379	0.313	< 0.0001**
	Prosthesis in the last 6 months	65	0.730	0.208	
	Complication after prosthesis	5	0.556	0.432	–

*BMI* Body mass index; *SD* Standard deviation; *WOMAC* Western Ontario and McMaster Universities Osteoarthritis Index

*P*-values represents the significance of differences between groups by means of ANOVA except for (*) *P*-value comparing with the whole sample’s utility value (0.520) (one sample *t*-test), and (**) *P*-value comparing sample before and after (6 months) the prosthesis (paired sample *t*-test)

Superscript letters indicate differences among the subgroups according to Bonferroni (region and number of comorbidities) or Tamhane’s T2 (WOMAC, OHS) post hoc tests for multiple comparisons at *P*-value< 0.01

### Results of the literature review and comparison with the primary study in Spanish population

Using the bibliographical search 116 references were identified of which 79 did not fulfill the inclusion criteria (Additional file 1: Figure S1). Of the 37 economic evaluations that did fulfill the inclusion criteria, 16 were not included in the CEA Registry or they were included but did not report utilities but rather QALYs and 6 included health states for which our database could not obtain utilities. Consequently, 15 papers were selected to extract health states and utilities, 10 in subjects with KOA [41–50], 4 in subjects with HOA [51–54], 1 in both [55]. These economic evaluations were performed in the USA (9), Canada (3), Germany (1), the United Kingdom (1), and Taiwan (1). Of these 15 papers, the CEA Registry included a total of 149 health states with their corresponding utilities. Using our Spanish database, it is not possible to estimate utilities for 61 out of the 149 health states. The main reason was that the health states were directly related to the consequences of joint replacement (complications, long-term outcomes) or with other technologies. Of the 88 states, a significant number of repeated health states-utilities pairs were identified because they were reported by the same authors using the same source [44, 45]. Therefore, a total of 51 pairs of health states-utilities (health states and their associated utilities) were selected: 45 pairs of health states-utilities for knee (Additional file 1: Table S4) and six pairs of health states-utilities for the hip (Additional file 1: Table S5). Several of these health states could be considered equivalent given their definition although there is heterogeneity in the definitions and/or values of utilities found in the literature. For example, three authors used in their models in KOA patients three apparently similar states of health but with clearly different utilities values: Osteoarthritis with conventional treatment (utility = 0.85) [50]; End stage knee osteoarthritis with treatment bridge (utility = 0.7) [46]; Conservative treatment (baseline) for knee osteoarthritis (utility = 0.55) [42]. Differences in these values may be for different reasons such as population characteristics or method used to estimate the utilities. In this specific case the first author used standard gamble, the second various bibliographic sources and the third author converted the EQ-5D VAS general population scores into standard gamble utilities. None of the studies explicitly used the EQ-5D index to estimate utilities.

Using our database, we can create and estimate utilities for 39 states approximately equivalent to those identified in the literature, 36 for knee (Additional file 1: Table S4) and 3 for hip (Additional file 1: Table S5). For 9 states it was not possible to estimate any utility because of lack of sample. A qualitative comparison between the values obtained by both sources shows that, in most cases (80%), the values obtained with our sample are lower than those identified in the literature. Among KOA patients, the largest difference between utilities obtained in the literature and utilities obtained in our sample occurred in health states for which a small sample size was obtained in our observational study. For those states with sample size of at least 30 subjects the largest difference (0.45 points) occurred in the state “0–1 comorbidity, age 65+, obese with osteoarthritis related pain’; the lower difference (0.005) and only one lower than 0.045 [40] occurred in the state ‘TKR during the last 6 months (WOMAC<60)’. Among patients with HOA, the largest difference (0.269 points) occurred in ‘severe HOA patients waiting for THR (0<OHS<19)’ [51] and the lowest difference (0.07) occurred in ‘THR during the last 6 months’ and only when we compare with one of the studies found in the literature [52].

## Discussion

This study aimed to obtain utilities by means of the EQ-5D-5 L questionnaire for different health states in KOA or HOA patients using an observational study performed in Spain and subsequently comparing these values with others obtained in international studies. Firstly, from our observational Spanish study, the average utility of samples with KOA and HOA were 0.544 and 0.520, respectively. The analysis of samples by subgroups revealed statistically significant differences according to level of pain, stiffness and physical function according to WOMAC and Oxford scales such that the worse the health condition the worse the utility. The validity of the instruments WOMAC [37] and OKH and OKS [30, 31] in Spain and their use in clinical practice support the use of these instruments to determine health states in economic evaluations. Consequently, the health state utility values obtained should be in mind for future economic studies.

To our knowledge, this is the first article whose aim was the characterization of utilities by health states in KOA and HOA from Spanish population by means of the most recent version of the EQ-5D questionnaire, the EQ-5D-5 L [11]. Previously the EQ-5D-5 L was used in the National Health Survey of Spain 2011/2012 [56]. In this survey it was observed that utility is worse as age increases and lower for women than men just as in our study. However, the values of utilities are far higher than ours because populations are very different. The National Survey questioned non-institutionalized persons (interviewed at home) who stated they had a diagnosis of arthrosis without specifying the joint involved.

Conversely, a review of utilities obtained from the Spanish population identified 6 prior studies focused mainly on joint replacement and in which the EQ-5D-5 L questionnaire was not used [11]. Three of them used the EQ-5D-3L [15–17]. One consisted of a prospective study in which the utility was evaluated before surgery (0.2 for knee and 0.47 for hip) and at 6 months (0.64 and 0.55 respectively, *n* = 40 for each estimate) [15]. In our study as expected there was a difference in the utility index before and after the prosthesis in both knee and hip, although values are substantially different to those obtained in our database probably because of the characteristics of the sample and differences in estimation between the 3 and 5 level version of the EQ-5D [57]. The psychometric qualities of the 5-level questionnaire defend its use in detriment to the 3-level questionnaire as the former is a more precise measure than the latter [57, 58]. Therefore, the average values of utilities obtained using our sample with the EQ-5D-5 L are more precise for characterization of health states in a future CUA study in comparison with values available up to now, apart from facilitating the necessary information (dispersion measures) to analyze the robustness of the model by means of a probabilistic sensitivity analysis.

Secondly, a literature review was carried out that aimed to compare international utilities with our utilities for similar health states. The first impression obtained from the literature review is the low number of publications in HOA patients in comparison to the literature available for KOA patients. The second impression is that, in general, utilities obtained in our observational study are lower than those collated in the literature. However, statistical comparisons between both were not conducted given that none of the studies identified in the review used the EQ-5D index, so a qualitative comparison was conducted and minimally relevant value according to McClure et al. was used [40]. It was possible to obtain and compare health states and utilities from our Spanish observational study for a significant number of health states and utilities used in international economic evaluations. Using Spanish utilities it could be possible to replicate all the models identified in the literature review except for two of them [45, 49]. One of them defined very specific conditions for which we do not have utilities for all of them [45]. Another evaluated among others the condition “Treatment of infection”, for which we only have one patient in our sample [49]. The qualitative analysis of the health states enables us to see the variety of forms in which an apparently same health state or condition can be defined [7]. These definitions, apart from the different methods to elicit the utilities and population/context differences [7], can be accounted for by differences in values found in literature and values estimated by ourselves. In any case, higher differences than the minimally relevant value according to McClure [40] were identified in almost 100% of cases. This shows the importance of appropriately choosing values of parameters in the models to avoid the economic evaluation being artificially affected [7].

Limitations of this study include those arising from the study design. First, a sample with broad criteria to recruit subjects with osteoarthritis leads, as expected, to a heterogeneous sample which necessarily is insufficient (in size) when we wish to characterize multiple population subgroups [59]. Consequently, the sample was heterogeneous in terms of treatments received and there was a small sample size to associate utilities with specific technologies such as prosthesis or medicines after surgery [59]. Second, the criteria used to define health states including cut-off points are at times arbitrary and other criteria may be necessary to define conditions in a future economic model [7]. Third, none of the studies included in the review used the EQ-5D-5 L questionnaire which is the one used in our observational study, whereby the differences observed should be taken with caution as they arise in part from different methods and instruments [7]. Four, the literature review was tackled pragmatically and with illustrative purposes, whereby it is under no circumstances an exhaustive review.

Finally, interesting analysis could not be performed. From a theoretic point of view, it would be desirable for economic evaluations performed in Spain to use values of utilities obtained by means of appropriate methods on a Spanish population. However, this is not always possible, and we must resort to review of literature reporting foreign population values [7]. This is the case of all the Spanish economic evaluations we have identified [24–26]. All of them used utilities from foreign studies in which EQ-5D was not used rather than other methods/instruments such as standard gamble or 15D questionnaire. These three studies evaluated the cost-effectiveness of prophylaxis medicines for the prevention of venous thromboembolism after TKR or THR [24–26]. Some health states in the studies were symptomatic deep-vein thrombosis or pulmonary embolism [24–26]. Because our study is observational and has a heterogeneous sample with short sample sizes for some subgroups [59], utilities could not be obtained for all these conditions related to post-surgery, whereby it was not possible to analyze the effect that using Spanish utilities would have meant.

The selection of utility values from foreign studies to be used in an economic evaluation model should be performed carefully taking care to ensure that the value chosen for the health condition is as real as possible but also making sure that the values used for all conditions considered in the economic model are coherent [7, 28, 59]. The main threat to this coherence is using data from different sources [7]. Therefore, observational or experimental studies that help to ascertain and characterize local populations are necessary [59]. Ideally, it should be possible to incorporate patient-report outcome measurements such as the EQ-5D-5 L into electronic clinical records [60] with the purpose of being able to routinely collate and use data that will be very useful for the economic evaluation of healthcare technologies [59]. Most authors have been interested in the cost-utility of total joint replacement [61] but these are not the only treatments, especially in early stages of osteoarthritis [20, 21]. Utility data estimated in our study may be of interest for researchers seeking to evaluate the cost-utility of these other alternatives in Spain.

## Conclusions

To sum up, the following conclusions can be drawn from the primary study and literature review. First, the worse the health condition in terms of level of pain, stiffness and physical function according to WOMAC and Oxford scales, the lower the utilities obtained by means of the EQ-5D-5 L questionnaire in Spain for both KOA and HOA. Secondly, this observational study offers values of utilities that to date were not available for a significant variety of health states. Thirdly, most utilities estimated with the Spanish sample are lower than those collated in the international studies identified. Finally, despite an observational study such as this having a broad sample this may not be sufficient to offer utilities for all possible health states related to existing technologies, it enables obtaining utilities directly from the local population which is necessary to help characterize and find out about these populations such that values of utilities used in future CUA performed in Spain are as close as possible to the country’s cultural reality.

## Supplementary information

**Additional file 1: Table S1.** Search strategy and results, NHS Economic Evaluation Database (NHS EED) by the Centre for Reviews and Dissemination (CRD), July 2017; **Table S2.** Utilities from the National Health Survey in Spain (2011–2012) and differences with observational Spanish study; **Table S3.** The most common EQ-5D-5 L health states according to Spanish observational study; Table S4. Comparison of health states and utilities from literature and from a Spanish study: knee osteoarthritis; **Table S5.** Comparison of health states and utilities from literature and from a Spanish study: hip osteoarthritis; **Figure S1.** Flow-chart of scoping review. (DOCX 64 kb)

## Data Availability

The datasets used or analyzed during the current study are available from the corresponding author upon reasonable request.

## Abbreviations

BMI: Body Mass Index
CUA: Cost-utility analysis
HOA: Hip osteoarthritis
HRQoL: Health-Related Quality of Life
KOA: Knee osteoarthritis
NSAID: Nonsteroidal anti-inflammatory drugs
OHS: Oxford Hip Score
OKS: Oxford Knee Score
QALY: Quality-adjusted life years
SD: Standard deviation
SYSADOA: Symptomatic slow acting drugs for osteoarthritis
THR: Total hip replacement
TKR: Total knee replacement
WOMAC: Western Ontario and McMaster Universities Osteoarthritis Index
