# Supplementary material for: EQ-5D-5 L utilities per health states in Spanish population with knee or hip osteoarthritis
Source: Health Qual Life Outcomes. 2019 Oct 30;17:164. doi: 10.1186/s12955-019-1230-x (PMC6822337; doi:10.1186/s12955-019-1230-x)
Supplement: Supplementary file 1 — Additional file 1: Table S1. Search strategy and results, NHS Economic Evaluation Database (NHS EED) by the Centre for Reviews and Dissemination (CRD), July 2017; Table S2. Utilities from the National Health Survey in Spain (2011–2012) and differences with observational Spanish study; Table S3. The most common EQ-5D-5 L health states according to Spanish observational study; Table S4. Comparison of health states and utilities from literature and from a Spanish study: knee osteoarthritis; Table S5. Comparison of health states and utilities from literature and from a Spanish study: hip osteoarthritis; Figure S1. Flow-chart of scoping review. (DOCX 64 kb) [file 12955_2019_1230_MOESM1_ESM.docx]

Additional file 1: Supplementary material

Title: EQ-5D-5L utilities per health states in Spanish population with knee or hip osteoarthritis

Authors: García Pérez, L et al.

Journal: Health Qual Life Outcomes

**Table S1. Search strategy and results, NHS Economic Evaluation Database (NHS EED) by the Centre for Reviews and Dissemination (CRD), July 2017**

| Search Number | Search | Hits |
| --- | --- | --- |
| 1 | (osteoarthritis) AND (knee) IN NHSEED | 92 |
| 2 | (osteoarthritis) AND (hip) IN NHSEED | 48 |
| 3 | #1 OR #2 | 116 |

**Table S2. Utilities from the National Health Survey in Spain (2011-2012) and differences with observational Spanish study**

| Subgroups | Diagnosed knee or hip osteoarthritis (source: our Spanish observational study) | | | General population (source: National Health Survey) | | | *P*-value (*) | Self-reported diagnosis of osteoarthritis (source: National Health Survey) | | |
| --- | --- | --- | --- | --- | --- | --- | --- | --- | --- | --- |
|  | N | Mean | Standard deviation | N | Mean | Standard deviation |  | N | Mean | Standard deviation |
| All | 750 | 0.533 | 0.287 | 20560 | 0.896 | 0.214 | <0.0001 | 5004 | 0.734 | 0.297 |
| <45 years | 14 | 0.600 | 0.345 | 7941 | 0.968 | 0.103 | 0.002 | 261 | 0.876 | 0.174 |
| 45-54.99 years | 54 | 0.510 | 0.292 | 3574 | 0.934 | 0.148 | <0.0001 | 563 | 0.824 | 0.227 |
| 55-64.99 years | 154 | 0.499 | 0.283 | 3171 | 0.892 | 0.201 | <0.0001 | 1034 | 0.797 | 0.246 |
| 65-74.99 years | 244 | 0.540 | 0.288 | 2731 | 0.858 | 0.218 | <0.0001 | 1260 | 0.773 | 0.250 |
| 75-84.99 years | 246 | 0.539 | 0.279 | 2341 | 0.756 | 0.296 | <0.0001 | 1402 | 0.683 | 0.306 |
| ≥85 years | 38 | 0.587 | 0.324 | 796 | 0.561 | 0.403 | 0.630** | 481 | 0.472 | 0.402 |
| Women | 463 | 0.522 | 0.284 | 11157 | 0.866 | 0.239 | <0.0001 | 3617 | 0.716 | 0.304 |
| Men | 287 | 0.550 | 0.292 | 9397 | 0.931 | 0.173 | <0.0001 | 1384 | 0.785 | 0.269 |

**P*-value comparing the mean of our study with the means from the National Health Survey (one sample *t*-test).

**Differences are also found when comparing the sample with knee *(P*=0.648) and hip (*P*=0.790) osteoarthritis separately with the mean of the general population.

**Table S3. The most common EQ-5D-5L health states according to Spanish observational study**

|  | Health state | Frequency | Percentage |
| --- | --- | --- | --- |
| Knee osteoarthritis | 32331 | 18 | 4.58% |
|  | 11121 | 15 | 3.82% |
|  | 33333 | 11 | 2.80% |
|  | 21121 | 8 | 2.03% |
|  | 21221 | 8 | 2.03% |
|  | 32231 | 8 | 2.03% |
| Hip osteoarthritis | 11111 | 15 | 4.20% |
|  | 33331 | 13 | 3.64% |
|  | 33332 | 11 | 3.08% |
|  | 21221 | 9 | 2.52% |
|  | 22222 | 9 | 2.52% |
|  | 21121 | 8 | 2.24% |
|  | 22231 | 8 | 2.24% |
|  | 33342 | 8 | 2.24% |

**Figure S1. Flow-chart of scoping review**

**Table S4. Comparison of health states and utilities from literature and from a Spanish study: knee osteoarthritis**

| From literature (CEA Registry^a^) | | | From our Spanish observational study | | |
| --- | --- | --- | --- | --- | --- |
| Health state | Utility weight | Reference | Health state^b,c^ | N | Utility weight (mean ± SD) |
| 0-1 comorbidity, age 25-44, non-obese with no osteoarthritis related pain | 0.995 | Losina 2013 | 0-1 comorbidity, age 25-44, non-obese with no osteoarthritis related pain | 1 | 0.713 |
| 0-1 comorbidity, age 25-44, non-obese with osteoarthritis related pain | 0.814 | Losina 2013 | 0-1 comorbidity, age 25-44, non-obese with osteoarthritis related pain | 0 | - |
| 0-1 comorbidity, age 25-44, obese with no osteoarthritis related pain | 0.921 | Losina 2013 | 0-1 comorbidity, age 25-44, obese with no osteoarthritis related pain | 0 | - |
| 0-1 comorbidity, age 25-44, obese with osteoarthritis related pain | 0.781 | Losina 2013 | 0-1 comorbidity, age 25-44, obese with osteoarthritis related pain | 0 | - |
| 0-1 comorbidity, age 45 - 64, non-obese with no osteoarthritis related pain | 0.952 | Losina 2013 | 0-1 comorbidity, age 45 - 64, non-obese with no osteoarthritis related pain | 15 | 0.726 ± 0.137 |
| 0-1 comorbidity, age 45-64, non-obese with osteoarthritis related pain | 0.806 | Losina 2013 | 0-1 comorbidity, age 45-64, non-obese with osteoarthritis related pain | 31 | 0.482 ± 0.257 |
| 0-1 comorbidity, age 45-64, obese with no osteoarthritis related pain | 0.918 | Losina 2013 | 0-1 comorbidity, age 45-64, obese with no osteoarthritis related pain | 9 | 0.818 ± 0.078 |
| 0-1 comorbidity, age 45-64, obese with osteoarthritis related pain | 0.773 | Losina 2013 | 0-1 comorbidity, age 45-64, obese with osteoarthritis related pain | 24 | 0.455 ± 0.261 |
| 0-1 comorbidity, age 65+, non-obese with no osteoarthritis related pain | 0.943 | Losina 2013 | 0-1 comorbidity, age 65+, non-obese with no osteoarthritis related pain | 48 | 0.793 ± 0.122 |
| 0-1 comorbidity, age 65+, non-obese with osteoarthritis related pain | 0.884 | Losina 2013 | 0-1 comorbidity, age 65+, non-obese with osteoarthritis related pain | 85 | 0.499 ± 0.216 |
| 0-1 comorbidity, age 65+, obese with no osteoarthritis related pain | 0.909 | Losina 2013 | 0-1 comorbidity, age 65+, obese with no osteoarthritis related pain | 17 | 0.777 ± 0.122 |
| 0-1 comorbidity, age 65+, obese with osteoarthritis related pain | 0.85 | Losina 2013 | 0-1 comorbidity, age 65+obese with osteoarthritis related pain | 83 | 0.425 ± 0.288 |
| 2-3 comorbidity, age 25-44, non-obese with no osteoarthritis related pain | 0.903 | Losina 2013 | 2-3 comorbidity, age 25-44, non-obese with no osteoarthritis related pain | 0 | - |
| 2-3 comorbidity, age 25-44, non-obese with osteoarthritis related pain | 0.721 | Losina 2013 | 2-3 comorbidity, age 25-44, non-obese with osteoarthritis related pain | 0 | - |
| 2-3 comorbidity, age 25-44, obese with no osteoarthritis related pain | 0.87 | Losina 2013 | 2-3 comorbidity, age 25-44, obese with no osteoarthritis related pain | 0 | - |
| 2-3 comorbidity, age 25-44, obese with osteoarthritis related pain | 0.688 | Losina 2013 | 2-3 comorbidity, age 25-44, obese with osteoarthritis related pain | 0 | - |
| 2-3 comorbidity, age 45-64, non-obese with no osteoarthritis related pain | 0.901 | Losina 2013 | 2-3 comorbidity, age 45-64, non-obese with no osteoarthritis related pain | 0 | - |
| 2-3 comorbidity, age 45-64, non-obese with osteoarthritis related pain | 0.713 | Losina 2013 | 2-3 comorbidity, age 45-64, non-obese with osteoarthritis related pain | 2 | 0.301 ± 0.183 |
| 2-3 comorbidity, age 45-64, obese with osteoarthritis related pain | 0.679 | Losina 2013 | 2-3 comorbidity, age 45-64, obese with osteoarthritis related pain | 0 | - |
| 2-3 comorbidity, age 45-64, obese with osteoarthritis related pain | 0.867 | Losina 2013 | 2-3 comorbidity, age 45-64, obese with osteoarthritis related pain | 3 | 0.151 ± 0.283 |
| 2-3 comorbidity, age 65+, non-obese with no osteoarthritis related pain | 0.891 | Losina 2013 | 2-3 comorbidity, age 65+, non-obese with no osteoarthritis related pain | 8 | 0.762 ± 0.173 |
| 2-3 comorbidity, age 65+, non-obese with osteoarthritis related pain | 0.791 | Losina 2013 | 2-3 comorbidity, age 65+, non-obese with osteoarthritis related pain | 21 | 0.432 ± 0.273 |
| 2-3 comorbidity, age 65+, obese with osteoarthritis related pain | 0.757 | Losina 2013 | 2-3 comorbidity, age 65+, obese with osteoarthritis related pain | 4 | 0.876 ± 0.088 |
| 2-3 comorbidity, age 65+, obese with osteoarthritis related pain | 0.858 | Losina 2013 | 2-3 comorbidity, age 65+, obese with osteoarthritis related pain | 19 | 0.417 ± 0.257 |
| 3+ comorbidity | 0.662 | Losina 2013 | 3+ comorbidity | 34 | 0.421 ± 0.303 |
| 3+ comorbidity, non-obese with no osteoarthritis related pain | 0.662 | Losina 2013 | 3+ comorbidity, non-obese with no osteoarthritis related pain | 3 | 0.787 ± 0.158 |
| Knee pain secondary to osteoarthritis | 0.69 | Nelson 2014 | KOA with pain | 286 | 0.455 ± 0.258 |
| Severe osteoarthritis | 0.69 | Losina 2013 | Severe KOA (0<OKS<19) | 155 | 0.327 ± 0.253 |
| All subjects with severe osteoarthritis | 0.69 | Losina 2014 |  |  |  |
| Pre-total knee replacement | 0.69 | Losina 2009 | KOA patients waiting for TKR | 92 | 0.4 ± 0.266 |
| End stage knee osteoarthritis | 0.6 | Mather 2014 |  |  |  |
| Baseline pre-treatment, UKA (unicompartmental knee arthroplasty) | 0.3 | Li 2013 (J Long Term Eff Med Implants) |  |  |  |
| Surgical treatment (baseline)for knee osteoarthritis | 0.53 | Li 2013 (Knee Surg Sports Traumatol Arthrosc) |  |  |  |
| Primary total knee arthroplasty | 0.92 | Novak 2007 | TKR during the last 6 months^d^ | 65 | 0.683 ± 0.243 |
| Primary total knee arthroplasty | 0.9 | Soohoo 2006 |  |  |  |
| Total knee arthroplasty in adult Canadian patients | 0.549 | Tso 2012 |  |  |  |
| Primary total knee arthroplasty | 0.9 | Mather 2014 |  |  |  |
| Treatment of infection | 0.5 | Soohoo 2006 | Infection related to TKR during the last 6 months^d^ | 1 | 0.794 |
| Post-total knee replacement western Ontario and McCaster (WOMAC) index <60 | 0.76 | Losina 2009 | TKR during the last 6 months (WOMAC<60)^d^ | 54 | 0.765 ± 0.142 |
| Post-total knee replacement western Ontario and McCaster (WOMAC) index >60 | 0.835 | Losina 2009 | TKR during the last 6 months (WOMAC≥60)^d^ | 11 | 0.279 ± 0.228 |
| Osteoarthritis with conventional treatment | 0.85 | Yen 2004 | KOA treated with conservative treatment for the last month (SYSADOA, pain medication, rehabilitation/physiotherapy) | 342 | 0.541 ± 0.267 |
| End stage knee osteoarthritis with treatment bridge | 0.7 | Mather 2014 |  |  |  |
| Conservative treatment (baseline) for knee osteoarthritis | 0.55 | Li 2013 (Knee Surg Sports Traumatol Arthrosc) |  |  |  |
| Knee injection for patients with osteoarthritis-based knee pain | 0.84 | Nelson 2014 | KOA treated with any type of injections^e^ | 46 | 0.579 ± 0.251 |
| Osteoarthritis with hyaluronan injections | 0.84 | Yen 2004 | KOA treated with hyaluronic acid^e^ | 9 | 0.663 ± 0.218 |

KOA: Knee osteoarthritis; OKS: Oxford Knee Score; SD: Standard deviation; SYSADOA: Symptomatic slow acting drugs for osteoarthritis; TKR: Total knee replacement.

^a^Health states and utilities weights obtained from CEA Registry.

^b^‘Pain’ is defined as moderate, severe or extreme pain according to pain/discomfort domain in EQ-5D-5L; ‘No pain’ is defined as slight or no pain according to pain/discomfort domain in EQ-5D-5L.

^c^‘Obese’ is defined as body mass index ≥30; ‘No obese’ is defined as body mass index <30.

^d^Estimated using the measurement at 6 months.

^e^Data collected directly from patients.

**Table S5. Comparison of health states and utilities from literature and from a Spanish study: hip osteoarthritis**

| From literature (CEA Registry^a^) | | | From our Spanish observational study | | |
| --- | --- | --- | --- | --- | --- |
| Health state | Utility weight | Reference | Health state | N | Utility weight (mean ± SD) |
| Severe osteoarthritis before hip resurfacing arthroplasty or total hip arthroplasty | 0.5 | Bozic 2010 | Severe HOA patients waiting for THR (0<OHS<19) | 62 | 0.231 ± 0.272 |
| Post primary total hip arthroplasty | 0.92 | Bozic 2010 | THR during the last 6 months^b^ | 65 | 0.73 ± 0.208 |
| Primary total hip arthroplasty | 0.8 | Cummins 2009 |  |  |  |
| Total hip arthroplasty in adult Canadian patients | 0.522 | Tso 2012 |  |  |  |
| Total hip replacement (traditional implant) including revisions | 0.964 | McKenzie 2003 |  |  |  |
| Complication after primary total hip arthroplasty surgery | 0.663 | Heintzbergen 2013 | Complication related to THR during the last 6 months^b^ | 8 | 0.782 ± 0.144 |

HOA: Hip osteoarthritis; OHS: Oxford Hip Score; SD: Standard deviation; THR: Total hip replacement.

^a^Health states and utilities weights obtained from CEA Registry.

^b^Estimated using the measurement at 6 months.
